# Role of phosphodiesterases in the pathophysiology of neurodevelopmental disorders

**DOI:** 10.1038/s41380-020-00997-9

**Published:** 2021-01-07

**Authors:** Sébastien Delhaye, Barbara Bardoni

**Affiliations:** 1 Université Côte d’Azur, CNRS UMR7275, Institute of Molecular and Cellular Pharmacology, 06560 Valbonne, France; 2 Université Côte d’Azur, Inserm, CNRS UMR7275, Institute of Molecular and Cellular Pharmacology, 06560 Valbonne, France

**Keywords:** Neuroscience, Psychiatric disorders

## Abstract

Phosphodiesterases (PDEs) are enzymes involved in the homeostasis of both cAMP and cGMP. They are members of a family of proteins that includes 11 subfamilies with different substrate specificities. Their main function is to catalyze the hydrolysis of cAMP, cGMP, or both. cAMP and cGMP are two key second messengers that modulate a wide array of intracellular processes and neurobehavioral functions, including memory and cognition. Even if these enzymes are present in all tissues, we focused on those PDEs that are expressed in the brain. We took into consideration genetic variants in patients affected by neurodevelopmental disorders, phenotypes of animal models, and pharmacological effects of PDE inhibitors, a class of drugs in rapid evolution and increasing application to brain disorders. Collectively, these data indicate the potential of PDE modulators to treat neurodevelopmental diseases characterized by learning and memory impairment, alteration of behaviors associated with depression, and deficits in social interaction. Indeed, clinical trials are in progress to treat patients with Alzheimer’s disease, schizophrenia, depression, and autism spectrum disorders. Among the most recent results, the application of some PDE inhibitors (PDE2A, PDE3, PDE4/4D, and PDE10A) to treat neurodevelopmental diseases, including autism spectrum disorders and intellectual disability, is a significant advance, since no specific therapies are available for these disorders that have a large prevalence. In addition, to highlight the role of several PDEs in normal and pathological neurodevelopment, we focused here on the deregulation of cAMP and/or cGMP in Down Syndrome, Fragile X Syndrome, Rett Syndrome, and intellectual disability associated with the *CC2D1A* gene.

## Introduction

### The family of phosphodiesterases

**Fig. 1:**
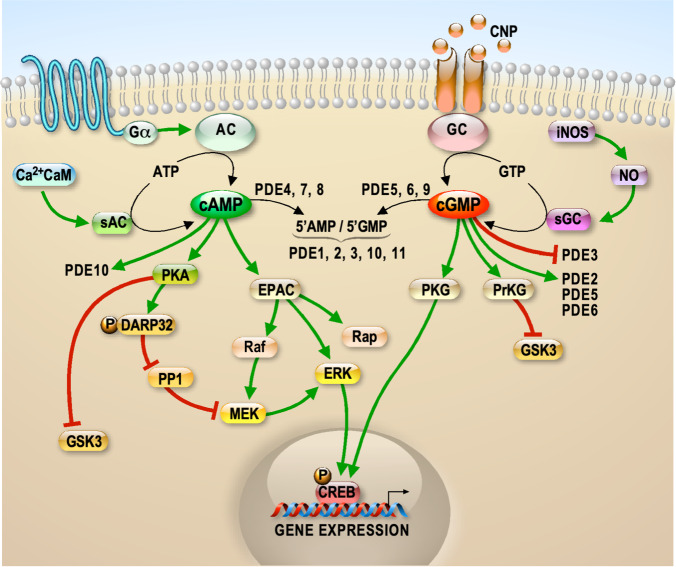
Neuronal pathways involving PDEs. Schematic of the regulation of cAMP synthesis by Adenylate Cyclases (AC) or s(soluble)AC and of cGMP synthesis by guanylate cyclases (GC) or s(soluble)GC. Specific degradation of cAMP and cGMP is catalyzed by various PDEs whose specificity is shown. Targets of cAMP and cGMP are shown, as well as well-known neuronal pathways involved in neurodevelopment. Red arrows indicate inhibition while green arrows indicate activation. PKA cAMP-dependent Protein Kinase, Ca^2+^CaM Ca^2+^/calmodulin-dependent protein kinase II, EPAC Exchange Protein directly Activated by cAMP, Rap Ras-related protein, ERK Extracellular signal-Regulated Kinase, Raf rapidly accelerated fibrosarcoma, DARP32 dopamine- and cAMP-regulated neuronal phosphoprotein, PP1 protein phosphatase-1, MEK MitogEn-activated protein kinase Kinase, PKG cGMP-dependent protein kinase, PrKG protein kinase, cGMP-dependent, GSK3 glycogen synthase kinase 3, iNOS inducible nitric oxide synthase; NO nitric oxide, CREB cAMP response element-binding protein, CNP C-type natriuretic peptide.

**Fig. 2:**
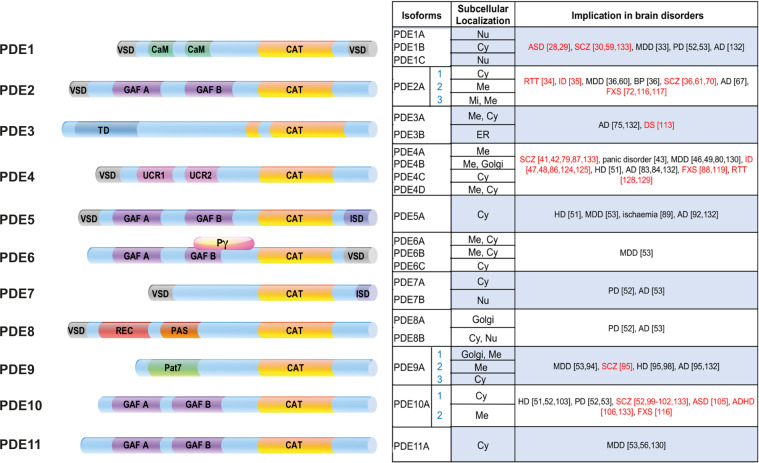
Structure, localization and implication of PDEs in brain disorders. Left panel. The structure of each PDE subfamily is indicated by representing the various domains. Catalytic domain providing the substrate specificity: CAT. Regulatory domains: GAF: the name is related to the proteins it is found in: cGMP-specific phosphodiesterases, adenylyl cyclases and the bacterial transcription factor FhlA. Ca^2+^CaM Ca^2+^/calmodulin binding site; PAS Per-ARNT-Sim domain, that is a structural motif. TD transmembrane domain. REC cheY-homologous receiver domain. UCR upstream conserved region. ISD isoform specific domain. Regions that are submitted to alternative splicing have been indicated as VSD variant-specific domain. This domain is called PAT7 in PDE9A variants [148] Pγ is part of the PDE6 holoenzyme in rod. Isoforms originated by different genes exist and are indicated for the following subfamily: PDE1 (A, B, and C), PDE3 (A and B), PDE4 (A, B, C and D), PDE6 (A, B and C), PDE7 (A and B) and PDE8 (A and B). PDE2, PDE5, PDE9, PDE10 and PDE11 subfamilies are represented by only one member, namely PDE2A, PDE5A, PD9A, PDE10A and PDE11A. Splicing variants (in blue) of the same protein are indicated when they determine a specific subcellular localization. Middle panel. Localization of each isoform and variant is indicated [4, 6, 148]. Cy Cytosol, Me membrane; Mi mitochondria; Nu Nucleus. Right panel. Brain disorders involving the various PDEs are indicated according to all the genetic and pharmacological information considered in the text. Neurodevelopmental disorders are highlighted in red. AD Alzheimer disease; ASD autism spectrum disorder; BP bipolar disorder; DS down syndrome; HD Huntington disease; ID intellectual disability; FXS fragile X syndrome; MDD major depression disorder, RTT Rett syndrome, SCZ schizophrenia.

Cyclic adenosine monophosphate (cAMP) and cyclic guanosine monophosphate (cGMP) are second messengers that regulate a variety of signaling pathways via direct interaction with cAMP-dependent protein kinase A (PKA) and cAMP-dependent protein kinase G (PKG), respectively [1]. Adenylate and guanylate cyclases (AC and GC) catalyze the synthesis of cAMP and cGMP starting from ATP and GTP, respectively. In the brain, activation of AC is mediated by heterotrimeric G proteins upon activation of G protein-coupled receptors (GPCRs) by extracellular stimuli (Fig. 1), while soluble AC is directly activated by Ca^2+^ [2] (Fig. 1). In the brain, soluble GC is mainly activated by nitric oxide (NO) and transmembrane GC is activated by C-type natriuretic peptide (CNP) [3] (Fig. 1). cAMP and cGMP are hydrolized by phosphodiesterases (PDEs). The enzymatic activity of PDEs was used as one of the initial processes providing evidence for the physiological importance of cAMP. Today, we know that PDEs catalyze the hydrolysis of the 3′ phosphate bond of cAMP and cGMP to generate 5′ AMP and 5′ GMP, respectively [1]. Mammalian PDEs are classified in 11 subfamilies of proteins encoded by 21 different genes, each one displaying multiple splice variants. These spliced variants often have different subcellular localization (Fig. 2) [1, 4]. PDEs are divided into three groups based on their specificity to cyclic nucleotides: specific to cAMP (PDE4, PDE7, and PDE8), specific to cGMP (PDE5, PDE6, and PDE9), and hydrolyzing both cAMP and cGMP (PDE1, PDE2, PDE3, PDE10, and PDE11). The PDEs in the last group have a higher affinity for one of the two cyclic nucleotides [1, 4]. This specificity is associated with a “glutamine switch”, a highly conserved glutamine residue that regulates the binding of the cyclic nucleotide purine ring in the binding domain [5]. The structural features of these enzymes, their regulatory domains, and catalytic regions are common among isoforms and are highly conserved across species. In each subfamily, the main variable regions between each member are the N- and C-terminal domain-containing elements for subcellular localization that is a critical element to define the specific function of each PDE [4, 6]. In Fig. 2, the 11 subfamilies are shown and for each one the functional domains are presented. Moreover, the activity of some PDEs depends on cGMP (PDE2, PDE3, PDE5, and PDE6), while cAMP activates PDE10A [1] (Fig. 1). PDEs are expressed in the cells of all tissues. The precise spatiotemporal expression of PDEs is crucial for the accurate regulation of cAMP and cGMP levels. Indeed, all PDEs appear to be expressed in the brain according to http://mousebrain.org/celltypes/ and each PDE displays a specific expression pattern, as illustrated in Supplementary Table I [7]. A study by Lakics et al. compared the expression levels of PDEs in different human brain regions [8]. In Supplementary Table II, we summarize the results of this study indicating the 2–3 most highly expressed PDEs in each of the analyzed brain regions. The expression of PDEs during brain development is poorly studied. In Supplementary Table III, we summarized the studies on this subject reported on Mouse Brain Atlas https://mouse.brain-map.org/static/atlas.

### Role of PDEs in the brain

In the brain, both cAMP and cGMP are essential during neurodevelopment as well as in maintaining synaptic plasticity, and ultimately in learning and memory [9–11]. Indeed, it has been reported that the levels of both cAMP and cGMP have critical roles in axon elongation and guidance [12, 13] and in regulating the morphology and growth of dendritic spines, where they have opposite effect [12]. cAMP abundance coupled to PKA signaling is critical to modulate assembly/disassembly/priming/recycling of neurotransmitter vesicles and, consequently, for synaptic transmission and plasticity events [14]. cGMP signaling can be transmitted through cyclic nucleotide-gated or hyperpolarization-activated cyclic nucleotide-gated ion channels. Furthermore, pharmacological inhibition of soluble GC or PKG slowed down the rate of recycling as well as endocytosis of synaptic vesicles. Indeed, the NO/cGMP/PKG pathway is known to modulate transmitter release and long-term changes of synaptic activity in various brain regions [15]. Overall, the balance between cAMP and cGMP levels is considered to be essential for shaping neuronal circuits [16]. Both cAMP- and cGMP-dependent signaling have been involved in neuronal migration [17]. In particular, a recent study suggested that primary cilium and centrosome integrate the cAMP signaling to favor neuronal migration [18]. Signal transduction starts when cAMP and cGMP induce the activation of PKA and PKG, respectively. Hence, PKA and PKG phosphorylate key proteins of a large array of downstream pathways. As an example, Fig. 1 illustrates the role of PKA and PKG in pathways involved in learning and memory (e.g., cAMP response element-binding protein (CREB) [19]) and in a large number of neurodevelopmental and psychiatric disorders (e.g., GSK3) [20]. Furthermore, cAMP activates the exchange protein directly activated by cAMP (EPAC), which regulates kinases (e.g., ERK and Raf) and Rap GTP-binding protein, a small-GTPase similar to Ras. These factors have key roles in modulation of molecular signaling involved in various aspects of neuronal differentiation, like the establishment of neuronal polarity or axonal growth and cone movement [21–24] (Fig. 1).

#### Genetic evidence of the implication of PDEs in neurodevelopmental disorders

Recent large-scale genome-wide association studies (GWAS) conducted by the Science Genetic Association Consortium [25] linked genetic variation in some PDE genes with multiple measures of human cognitive function. Single-nucleotide polymorphisms (SNPs) found in *PDE1C, PDE2A, PDE4B*, and *PDE4D* reached genome-wide significance with educational attainment and cognitive performance [26]. Of note, in this study, the highest statistical significance was reached by SNP rs72962169 in the *PDE2A* gene for GWAS/Educational attainment as measured by the highest level of education achieved (Eduyears), conjoint analysis/Eduyears, and multi-trait analysis/Eduyears [25]. Starting from this information, we considered here the genetic evidence in patients and in transgenic animals (See also Supplementary Table IV) supporting the implication of PDEs in neurodevelopmental brain disorders.

Three *PDE1* genes *(PDE1A*, *PDE1B*, and *PDE1C*) have thus far been identified. Enzyme activity is increased by Ca^2+^/calmodulin binding to the regulatory site located in the N-terminal region of the enzyme [27]. An intronic SNP in the *PDE1C* gene was found to be associated with autism spectrum disorder (ASD; *p* value < 1.0E−04) in a GWAS meta-analysis of 7387 ASD cases and 8567 controls [28]. Inherited missense variants in *PDE1B* gene have also been identified in probands with ASD [29] and with schizophrenia (SCZ) [30]. The knockdown of *Pde1b* in the hippocampus enhances spatial and contextual memory [31]. Conversely, *Pde1b*-knockout (KO) mice display locomotor hyperactivity, spatial learning deficits [32], and antidepressant-like phenotypes [33]. These findings indicate the need to tightly modulate *Pde1b* expression levels by pharmacological approaches to observe an impact on socio-cognitive behavior.

PDE2A is a dual-specific PDE that breaks down both cAMP and cGMP and is activated by cGMP. A homozygous splicing mutation in *PDE2A* was found in two patients with atypical Rett syndrome (RTT), displaying neurodevelopmental delay [34], while a homozygous loss-of-function mutation in *PDE2A* was associated with early-onset hereditary chorea-predominant movement disorder and intellectual disability (ID) [35]. Patients with bipolar disorder showed reduced *PDE2A* mRNA levels in the hippocampus, caudal entorhinal cortex, and striatum, while patients with SCZ, bipolar disorder, and major depressive disorder (MDD) showed reduced *PDE2A* mRNA levels in the amygdala. Furthermore, patients with schizophrenia have reduced *PDE2A* mRNA levels in the frontal cortical region [36]. *Pde2a*-KO mice have been generated but they are lethal during embryogenesis due to cardiac failure [37]. To our knowledge, no studies have been performed on heterozygous animals so far.

The *PDE4* family comprises four genes, *PDE4A–D*, each expressed as multiple variants. The selective modulation of individual *PDE4* subtypes revealed that individual subtypes exert unique and non-redundant functions in the brain [38]. The role of *PDE4* in learning and memory has been studied extensively in the *Drosophila* fruit fly, where only one homolog of the family known as *dunce* (*dnc*) is present, and in mice. Mutational studies in flies have identified PDE4 as a key modulator of a signaling pathway critical for associative learning, courtship behavior, and neurodevelopment. Deletion of the *dnc* gene in *Drosophila* disrupts learning and memory by preventing the hydrolysis of cAMP, thereby altering the normal spatial and temporal patterning of cAMP signaling [39, 40].

PDE4B interacts directly with DISC1, which is a well-known genetic risk factor for mental illness. Mutations in the *DISC1* and *PDE4B* genes reduce the association between both proteins, reducing PDE4B activity, which has been shown to be correlated with SCZ [41]. In particular, it was reported that an SNP in *PDE4B* conferred a protective effect against SCZ in women [42]. The implication of the *PDE4B* gene in behavior and mood is also supported by the association of *PDE4B* gene polymorphisms with protection from panic disorder in Russians subjects [43]. *Pde4b*-KO mice show a significant reduction in prepulse inhibition and an exaggerated locomotor response to amphetamine correlated with decreased striatal dopamine and serotonin activity [44]. *Pde4b*-KO mice display enhanced early long-term potentiation following multiple induction protocols, and they exhibit significant behavioral deficits in associative learning using a conditioned fear paradigm [45]. In addition, they display decreased head-dips and time spent in head-dipping in the hole-board test, reduced transitions and time on the light side in the light–dark transition test, and decreased initial exploration and rears in the open-field test were observed [46]. Overall, these studies suggest that PDE4B is involved in signaling pathways that contribute to anxiogenic-like effects on behavior [46]. *PDE4D* mutations have been reported as causative for acrodysostosis type 2 with or without hormone resistance (ACRDYS2), a disorder characterized by severe ID [47, 48], leading to the conclusion that altered PDE4D levels may affect brain functioning. *Pde4d*-KO mice exhibit decreased immobility in tail suspension and forced swim tests [49], suggesting that PDE4D may play a role in the pathophysiology and pharmacotherapy of depression.

PDE10A is a dual-specific PDE that breaks down both cAMP and cGMP. *Pde10-*KO mice display a modest impairment of latent inhibition, a decrease in exploratory locomotor activity, and a delay in the acquisition of conditioned avoidance responses. Furthermore, a decrease in spontaneous firing in striatal medium spiny neurons and a significant change in striatal dopamine turnover, which is accompanied by an enhanced locomotor response to amphetamines, was observed in these mice [50]. All these data support the strong implication of PDE10A in striatal function, and thus its possible involvement in the pathophysiology of disorders such as Huntington (HD) and Parkinson disease (PD) and SCZ [51–53] (see below).

PDE11A is also a dual-specific PDE that breaks down both cAMP and cGMP. *PDE11A4*, one of the four *PDE11A* splice variants, is involved in the hippocampal formation in humans and rodents, and is highly enriched in the rodent ventral hippocampal formation compared to the dorsal hippocampal formation [54]. *Pde11A*-KO mice show enlarged lateral ventricles and increased activity in CA1, altered formation of social memories and abnormal stabilization of mood [54, 55]. This latter conclusion is also supported by other authors associating *PDE11A* with both MDD and response to antidepressant drugs [56]. Indeed, 16 SNPs in *PDE11A* were studied in patients affected by MDD and sharing 18 common haplotypes. Six haplotypes showed significantly different frequencies between the MDD group and the control group. Furthermore, in patients treated with two different antidepressant drugs—increasing the cGMP/cAMP ratio [57, 58]—the frequency of one haplotype was significantly lower in the remitter group than in the nonremitter group [56].

#### Pharmacological modulation of PDEs

Similar to the generation of animal models, the identification of specific and powerful inhibitors of PDEs allowed studying the functional role of these enzymes, paving the way for their use in the clinic.

A well-known inhibitor of PDE1B is DSR-141562, which inhibits locomotor hyperactivity and reverses social interaction and novel object recognition in normal mice and rats. This molecule was also shown to improve cognition in the marmoset, a non-human primate [59] by acting through CREB and DARPP32 [32, 59] (Fig. 1). DSR-141562 has been proposed as a therapeutic candidate for positive, negative, and cognitive symptoms in schizophrenia [59].

Many specific PDE2A inhibitors have been generated and characterized. Hcyb1 produces neuroprotective and antidepressant‐like effects in mice [60]. TAK-915 shows efficacy in ameliorating cognitive and social impairment in induced rat models of SCZ [61] and it improves cognitive impairment associated with aging in mice [62]. To date, one phase I clinical trial has been reported for this compound (Supplementary Table V). A clinical trial has been carried out using a specific PDE2A inhibitor by Pfizer to treat migraine, but the results have not been communicated to the scientific community (Supplementary Table V). This study suggests a possible role of PDE2A in pain control. This hypothesis is supported by a study reporting that Bay 60–7550, another well-known inhibitor of PDE2A, alleviates radicular inflammation and mechanical allodynia in a rat model of non-compressive lumbar disc herniation [63]. Bay 60–7550 is likely the most studied PDE2A inhibitor, probably due to its commercial availability. It has been reported to have anxiolytic properties and to ameliorate learning, memory, and synaptic plasticity in normal animals [64–66] and in neurodegeneration models [67, 68]. This drug could also be used for treatment of alcoholism since it was reported to decrease ethanol intake and preference in mice [69]. Despite all these results, no clinical trials testing Bay 60–7550 have been reported, suggesting possible toxicity of this compound. Controversial results concerning the poor ability to cross the blood-brain barrier after oral administration to adult rats [64, 70] and the efficacy of this drug in treating brain disorders when used by intraperitoneal injection in adult and infant mice and rats, might suggest that the bioavailability of Bay 60–7550 in the brain is dependent on the route of administration and/or by the age of the treated subject [65, 71, 72]. PDE2A has been also involved in the pathophysiology of FXS (see below).

Cilostazol is the most studied PDE3-specific inhibitor already used in clinics for the treatment of symptoms of intermittent claudication due to its vasodilator and antiplatelet actions [73]. A clinical trial in patients with mild cognitive impairment is in progress using this compound (Supplementary Table V). This new potential use of cilostazol is based on retrospective study showing that patients using this drug daily as an antiplatelet drug have a decreased risk of developing dementia [74] and reduces the decline in cognitive function in patients with stable AD [75]. In the hippocampus, Cilostazol increases the levels of c-fos and of insulin-like growth factor 1 (IGF-1) [76] and activates CREB in PC12 cells [77].

Several clinical trials have been carried out using roflumilast, a highly specific PDE4 inhibitor. Double-blind studies have shown that acute treatment with roflumilast improves verbal memory performance in elderly and healthy participants [78] and in patients with SCZ [79]. FCPR16 also shows antidepressant-like effects in mice exposed to chronic unpredictable mild stress [80]. Rolipram is another well-known PDE4 inhibitor, whose utilization results into the increased phosphorylation of CREB [81]. Consistently, a single injection of rolipram improves spatial memory deficits in aged mice [82] and memory consolidation of conditioned fear [81]. Furthermore, in the model of Amyloidβ-induced memory impairment, mimicking AD, both treatments with rolipram or BPN14770 improve memory performances [83–85]. Rolipram abolishes long-term memory defects in a mouse model of Rubinstein-Taybi syndrome, caused by the presence of a truncated form of CREB [86] and has antipsychotic properties [87]. This drug was used to improve the phenotype of both fly and mouse models of FXS [88].

PDE5 inhibitors are essential for the vascular effects and treatment of erectile dysfunction. However, PDE5A inhibitors have been implicated in memory function in various studies. In rats, sildenafil, an inhibitor of PDE5, promotes neurogenesis, reduces neurological deficits, and promotes functional recovery after stroke and focal cerebral ischemia [89]. In addition, this drug improves cognition and spatial learning [90, 91]. Sildenafil has been shown to produce long-lasting amelioration of synaptic function, CREB phosphorylation and to increase Brain-Derived Neutrophic Factor (BDNF) levels [92]. In humans, sildenafil affects selective auditory attention and verbal recognition memory [93]. In particular in AD patients, a single dose of this drug increased cerebral blood flow and the cerebral metabolic rate of oxygen [92].

WYQ-C36D, a high-affinity PDE9A inhibitor, produces antidepressant-like, anxiolytic-like, and memory-enhancing effects in stressed mice [94]. Inhibition of PDE9A in rats with PF-4447943 and PF-4449613 demonstrated the implication of this PDE in tasks depending on hippocampal cholinergic function and sensory gating. These results suggested the utilization of PDE9A inhibitors to treat AD, SCZ, or HD [95]. However, two double-blind randomized controlled phase II studies using two different inhibitors of PDE9A, namely BI 409306 or PF-04447943, failed to prove efficacy in improving cognition in patients with AD [96, 97]. Another clinical trial testing the effect of BI 409306 on SCZ patients is currently ongoing (Supplementary Table V). Recently, the treatment of a HD rat model with PF-4447943 suggested that this drug facilitates striatal cGMP signaling and glutamatergic cortico-striatal transmission. This could help to alleviate motor and cognitive symptoms associated with HD by restoring striatal function [98].

Data obtained by studying a mouse model supported the pivotal role of PDE10A in striatal signaling and striatum-mediated salience attribution, a process that is severely disrupted in patients affected by schizophrenia [99]. Indeed, TAK-063 (Balipodect) and T-251 dose-dependently suppress hyperactivity and improve cognitive deficits, respectively, in MK-801 mice, a model for acute psychosis [99, 100]. A phase II randomized and placebo-controlled clinical trial showed a potential beneficial effect of TAK-063 in subjects with acute exacerbation of SCZ [101]. Two clinical trials are currently testing another PDE10A inhibitor, Lu AF11167, for the treatment of negative symptoms in patients with SCZ (Supplementary Table V). These latter results are consistent with the observation that *Pde10a* mRNA is a target of miR-137, whose absence is associated with SCZ. Partial loss of miR-137 in heterozygous conditional KO mice results in increased PDE10A levels and in deregulated synaptic plasticity, repetitive behavior, and impaired learning and social behavior. Both treatment with papaverine, a PDE10A inhibitor, and knockdown of *Pde10a* ameliorate the deficits observed in the miR-137 mouse model [102]. PDE10 inhibition increases the expression of BDNF and the phosphorylation of both CREB and the alpha-amino-3-hydroxy-5-methyl-4-isoxazole propionate (AMPA) receptor GLUA1 in a mouse model of HD [103]. Of note, the implication of this receptor in neurodevelopmental disorders is well known [104]. Indeed, recently, papaverin has been reported to attenuate neurobehavioral abnormalities in a rat model of ASD [105] and to ameliorate hyperactivity, inattention and anxiety in a model of attention deficit hyperactivity disorder (ADHD) [106].

#### Altered activity of PDEs in neurodevelopmental disorders—preclinical conclusion and translational elements

Down syndrome (DS) is the most frequent form of ID (1:600 newborns) and is caused by an extra copy of chromosome 21. A specific region on this chromosome, namely the DS critical region, is necessary and sufficient to produce the main phenotype of DS: cognitive congenital malformations (particularly cardiovascular) and dysmorphic features. Immune disturbances in DS account for autoimmune alopecia, autoimmune thyroiditis and leukemia, respiratory tract infections, and pulmonary hypertension [107]. Several genes in triple dosage have been reported to contribute to ID in patients with DS. The most studied is the *dual-specificity tyrosine phosphorylation-regulated kinase 1A (DYRK1A*) gene encoding a kinase involved in the formation and maturation of dendritic spines from dendrites [108]. Mutations in *DYRK1A* have been found in patients affected by ASD [109]. DYRK1A has CREB among its targets [110], whose activity is regulated by both cAMP and cGMP (Fig. 1), thus suggesting a clear interference of deregulated expression of DYRK1A in cAMP and/or cGMP pathway in DS. Another factor with elevated expression levels in DS is the amyloid precursor protein. This deregulated expression is likely the cause of β-amyloid (Aβ) plaques that are present in young patients with DS and represent one of the neuropathology hallmarks shared between DS and AD [111]. As reported in Supplementary Table V, several clinical trials to treat AD with PDE inhibitors are currently ongoing. Hence, this information, together with reduced levels of cAMP reported in one of the most-widely used models of DS, the Ts65Dn mouse [112], pushed some authors to study the implication of PDE activity in the pathophysiology of DS. In a recent study, the administration of cilostazol (PDE3 inhibitor) to Ts65Dn mice from fetal to adult age ameliorated cognition and sensorimotor function in females. The same treatment in males improved their hyperactive locomotion and spatial memory [113]. Overall, these results seem to be consistent with the previous observation that cilostazol promotes the clearance of Aβ plaques and rescues cognitive deficits in a mouse model [113] of AD and reduces the cognitive decline in patients affected by AD [75].

*FXS* is caused by the silencing of the *fragile X mental retardation gene* (*FMR1*). FXS is the most common form of inherited ID (1:4000 males and 1:7000 females). Patients might also display hyperactivity, attention deficit, ASD, language problems, and seizures [114]. Deregulation of cAMP was one of the first molecular hallmarks defined in FXS patients [115]. *FMR1* encodes the fragile X mental retardation protein (FMRP), an RNA-binding protein that is highly expressed in all brain regions in both neurons and glial cells and mainly involved in translational regulation, being both a repressor and an enhancer of translation [116]. Among the mRNA targets of FMRP, three PDEs, *PDE1A*, *PDE2A*, and *PDE10A*, have been identified [116]. The expression of PDE2A in the cortex and hippocampus is negatively modulated by FMRP both on a translational level and by affecting dendritic transport of encoding mRNAs [116]. Consistently, PDE2A expression levels are elevated in *Fmr1*-KO brains, resulting in reduced levels of both cAMP and cGMP. Treatment of *Fmr1*-KO mice with Bay 60–7550 normalizes the social and cognitive deficits of infant and adolescent *Fmr1*-KO mice, increased the maturity of axons and dendritic spines of *Fmr1*-KO neurons and normalizes the exaggerated hippocampal m-GluR5 long-term depression of *Fmr1*-KO CA1 [72]. Inhibition of PDE2A activity by Bay 60–7550 rescues the release of synaptic vesicles, which is reduced in the absence of FMRP [117]. Consistently, PDE2A is the only PDE associated with docked vesicles [118]. Collectively these findings suggest the implication of PDE2A in the release of neurotransmitters. Another target of FMRP is the mRNA encoding *Pde10a*, whose expression is likely increased in *the Fmr1*-KO mouse brain [116]. Balipodect, an inhibitor of PDE10A, has been accepted by the European Medicine Agency as a potential treatment for FXS (EMADOC-628903358-742). The specific inhibition of PDE4D by BNP14770, a drug developed by Tetra Therapeutics, was shown to be potentially useful for the treatment of FXS. While rolipram—already used to treat FXS animal models [88] inhibits all subtypes of PDE4, BPN14770 is selective for PDE4D. BNP14770 inhibits the enzyme by closing one of the two upstream conserved regions at the regulatory domains across the active site, which limits cAMP access. Daily treatment of *Fmr1*-KO mice for 14 days with BNP14770 improves social interaction and natural behaviors (such as nesting and marble burying), and rescues the altered dendritic spine morphology of *Fmr1*-null neurons [119]. The details of two clinical trials for this molecule are shown in Supplementary Table V. Positive results obtained in the phase II clinical trial with BNP14770 and enrolling 30 FXS adult male subjects (age 18–41 years) were recently announced: https://www.fraxa.org/positive-results-reported-in-phase-ii-fragile-x-clinical-trial-of-pde4d-inhibitor-from-tetra-therapeutics/. It was reported that BPN14770 antagonizes the amnesic effects of scopolamine, increases cAMP signaling in the brain, and increases BDNF and markers of neuronal plasticity associated with memory [85]. This might suggest that this drug will be beneficial to other forms of ID and ASD.

ID associated with the *coiled-coil and C2 domain-containing 1A (CC2D1A)* gene. Functional loss of *CC2D1A* causes a rare form of autosomal recessive ID, sometimes associated with ASD and seizures [120]. While in *Drosophila*, the loss of the ortholog of *CC2D1A, lgd*, is embryonically lethal [121], *Cc2d1a* conditional KO mice display deficits in neuronal plasticity and in spatial learning and memory, which are accompanied by reduced sociability, hyperactivity, anxiety, and excessive grooming [122]. The *CC2D1A* gene encodes a transcriptional repressor with essential functions in controlling synapse maturation. This protein regulates the expression of the 5-hydroxytryptamine (serotonin) receptor 1A gene in neuronal cells (HTR1A), likely playing a role in the altered regulation of HTR1A, that is known to be associated with SCZ, anxiety, and MDD [123]. CC2D1A is also localized in cytoplasm where it acts as a scaffold protein in the PI3K/PDK1/AKT pathway [124]. CC2D1A is an interactor of PDE4D regulating its activity and thereby fine-tuning cAMP-dependent downstream signaling [125]. Indeed, it regulates CREB activation in hippocampal neurons by increasing PDE4D activity only in male *Cc2d1a-*deficient mice. Consistently, the male mouse model for CC2D1A-associated disorder shows a deficit in spatial memory that can be restored by inhibiting PDE4D activity. *Cc2d1a*-deficient female mice do not display this phenotype and pharmacological treatment has no effect on their behavior [125].

*RTT* is a neurodevelopmental disorder that mostly affects girls (1:10000 newborns). In classical RTT, girls display an apparently normal development for 6–18 months before developing severe phenotypes characterized by language problems, deficits in learning and coordination, stereotypies, sleep disturbances, seizures, and breathing deficiencies. Overall, RTT is considered to have a characteristic clinical course of four stages: (I) early-onset stagnation, (II) developmental regression, (III) pseudostationary period, and (IV) late motor deterioration. RTT is caused by mutations in *Methyl CpG binding Protein 2 (MECP2)*, an X-linked gene encoding a transcription factor that binds methylated DNA and acts both as a repressor and enhancer of transcription [126]. The protein modulates a network of pathways, including the BDNF, PI3K, and ERK pathways [127]. Dysfunction of MECP2 affects morphology and density of dendritic spines, synaptic plasticity, and neuronal migration [126]. Furthermore, reduced cAMP levels were found in neurons of organotypic slices obtained from the brain of a mouse model of RTT due to increased activity of PDE4. Indeed, treatment with rolipram allowed normalization of the growth of neuronal processes and the cAMP transients evoked by electrical stimulation in preBötC neurons [128]. Consistently, reduced levels of CREB and phospho-CREB have been reported in neurons differentiated from human embryonic stem cells and from induced pluripotent stem cells lacking functional MECP2 and displaying an altered arborization. Rolipram rescued these cellular phenotypes as well as several behavioral phenotypes in female RTT mice [129].

## Discussion

The results obtained thus far indicate that modulation of PDE activity can be effective for disorders characterized by depressive behavior or memory deficits (Fig. 2) [130, 131]. Clinical trials have mostly addressed the treatment of AD [132] and schizophrenia [133], while two trials are in progress for a form of ASD and only one for depression (Supplementary Table V). We believe that understanding the role of PDE treatment in neurodevelopmental disorders, such as ID, ASD, or both is a very important result obtained from recent studies. Indeed, the pharmacological inhibition of two PDEs (PDE2A and PDE4D) has been associated with multiple autism-like behaviors and cognitive deficit at different ages in mouse models [116, 119, 125]. The inhibition of PDE10A has been proposed for FXS (even if no data have been published yet), ASD [105], and ADHD [106]. The inhibition of PDE3 has been used in DS and is potentially useful for ASD [113]. Indeed, this drug protects against cognitive impairment and white matter disintegration in a mouse model of chronic cerebral hypoperfusion [73], a phenotype characterizing some ASD patients [134]. It is also interesting to consider that low cAMP concentrations favor inflammation due to the increase in IL-8, IL-12, IL-17, IL-22, IL-23, tumor necrosis factor-α, γ-interferon, chemokine C–X–C motif ligand 9 (CXCL9), and CXC10 levels [135]. This suggests that PDE inhibitors are thought to have anti-inflammatory and neuroprotective effects. Increasing experimental evidence supports the presence of neuroinflammation as a relevant element of neurodevelopmental disorders [136, 137]. This indicates the importance of carrying out specific screenings of the expression levels of PDEs in cohorts of patients affected by idiopathic neurodevelopmental diseases such as ASD and/or ID. To achieve this, it could be useful to measure the levels of cAMP and/or cGMP in neurons derived from induced pluripotent stem cells or in extracellular vesicles from the central nervous system obtained from fibroblasts and blood, respectively, of these patients. The most accurate method to measure the dynamic levels of these second messengers in real-time is represented by the use of specific FRET-based cAMP (or cGMP) biosensors [18, 138]. This could help to establish therapeutic approaches with molecules modulating the activity of specific PDEs.

PDEs involved in various brain disorders so far (Fig. 2) are cAMP-specific, cGMP-specific or have a double target. This can raise the issue whether an altered cAMP/cGMP ratio could represent a pathophysiological element for these diseases. In this context, a good example is provided by the studies on depression showing that phenotypic improvement can be reached by changing the cGMP/cAMP ratio and then, paradoxically, both by increasing cGMP levels or by reducing cAMP levels [56–58, 130]. Moreover, also PDEs involved in FXS target both cAMP and cGMP [72, 116, 119] (Fig. 1). Indeed, PDE4 is a cAMP-specific isoform, while both cAMP and cGMP are targets of PDE10A and PDE2A, this latter enzyme being stimulated by cGMP (Fig. 1) [1]. PDE1A is activated by Ca^2+^ [1] and is involved in FXS [116], a disorder displaying a deregulated Ca^2+^ homeostasis in brain [139, 140]. We can speculate that an altered cAMP/cGMP ratio is a pathophysiological element in FXS and the modulation of one of those PDEs is sufficient to restore it. This could explain the results in preclinical tests [72, 119] and clinical assays (Supplementary Table V). Thus, it would be difficult to dissect the impact of each PDE in the neurological-psychiatric phenotypes present in FXS. This led to the conclusion that PDE transgenic animals are a main need in the field. Indeed, it is surprising that only a few PDEs have been extensively studied for their impact on brain function by using classical KO animal models. Here, we have reported those models displaying clear psychiatric or neurodevelopmental disorders. In the case of *Pde2a*, the KO phenotype has never been studied since homozygous mutant mice are not viable [37]. In other cases, a phenotype was not known or was never reported (e.g., PDE5). This latter example is very interesting because pharmacological inhibition of PDE5 resulted in potentiating neurogenesis and memory enhancement [89–92]. The use of pharmacological preclinical studies is critical to reach the clinical setting but is not enough to understand the role of PDEs during brain development. Indeed, these drugs are administrated only during the post-natal life and often after the weaning, thus missing the possibility to evaluate their impact in early steps of brain development and/or synaptogenesis. Furthermore, genetic silencing of specific PDEs’ variants could allow to study the modulation of cAMP and cGMP abundancy in subcellular compartments (Fig. 2). Studying such a large family of proteins is challenging due to their overlapping functions and because of their actions that are not brain specific. However, tools exist today (e.g., conditional KO and optogenetics) to conduct studies focused on the spatio-temporal role of each PDE in normal and pathological brain development and to elucidate their effects on socio-cognitive behaviors at different ages. These studies should also allow the identification of compensation activities by the various members of the PDE family, as suggested, for instance, by the observation that the expression levels of *Pde10a* mRNA are elevated in the striatum of *Pde1b-KO* mice [33].

It is critical that the effects of genes and pathways impacted by the various brain-expressed PDEs be studied in detail in various brain regions and at the synaptic level during brain development. These studies will result in the precise identification of the molecular pathways modulated by each PDE. For instance, an link between PDE3-dependent cAMP signaling with the IGF-1 pathway was established using the specific PDE3 inhibitor cilostazol [76]. Recombinant IGF-1 and some related compounds have emerged as potential therapeutics for the treatment of neurodevelopmental disorders [141]. Trofinetide is a neurotrophic peptide derived from IGF-1 that has a long half-life and is well tolerated. Chronic treatment of *Fmr1*-KO mice with trofinetide corrects learning and memory deficits, hyperactivity, and social interaction deficits displayed by these animals [142]. Thus, a phase II clinical trial for trofinetide was performed. After only 28 days of treatment, improvements in higher sensory tolerance, reduced anxiety, better self-regulation, and more social engagement were observed (NCT01894958). Interestingly, upon the sponsoring of ACADIA Pharmaceuticals Inc., a phase III clinical trial with trofinetide is in progress in 5–15 years old girls affected by RTT (NCT04181723). On the same way and consistent with previous results linking cAMP with BDNF transcription [143], inhibition of PDEs has been reported to increase BDNF levels [52, 53, 85, 92, 103], whose deregulation is involved in the pathophysiology of depression [144], SCZ [133, 145], RTT [126, 146], and FXS [147]. Therefore, we conclude that these findings support the idea of future therapies for psychiatric disorders at the crossroads of pathways involving cAMP and/or cGMP and neurotrophic factors.

Finally, the surprising results from Zamarbide et al. (CC2D1A) and Tsuji et al. (DS) suggest the possibility that a sex-specific regulation (or deregulation) of cAMP or cGMP levels exists [113, 125]. These findings are particularly important for the treatment of ASD and ID, since the ratio of incidence of this disorder is 3:2 in males vs. females, and this could result in a sex-specific treatment. This possibility should be considered for future studies on PDEs, reinforcing the need to study their relevance for PDE action on brain development and functioning at various ages when sex hormones are present (or not) and they can affect PDE-related pathways differently.

## Supplementary information

Supplementary Table I

Supplementary Table II

Supplementary Table III

Supplementary Table IV

Supplementary Table V
