# Supplementary material for: Role of phosphodiesterases in the pathophysiology of neurodevelopmental disorders
Source: Mol Psychiatry. 2021 Jan 7;26(9):4570–82. doi: 10.1038/s41380-020-00997-9 (PMC8589663; doi:10.1038/s41380-020-00997-9)
Supplement: Supplementary file 1 — Supplementary Table I [file 41380_2020_997_MOESM1_ESM.docx]

**Supplementary Table I. PDEs expression in specific adult mouse brain cell type**

| **PDEs** | **Region Expression** | **Cluster Expression** |
| --- | --- | --- |
| PDE1A | - CC - Tlc - Hyp | - Excitatory neurons TEGLU2:24 (Marker gene of TEGLU10) - Cholinergic interneurons TECHO - Cholinergic neurons DECHO1 |
| PDE1B | - St - Hb | - D1/D2 medium spiny neurons MSN1:4 - Excitatory neurons HBGLU4:8 |
| PDE1C | - DRG - OB - Med | - Peripheral Sensory Neurons PSNF1, PSNP1-4-5 - External plexiform layer interneuron OBINH6 (Marker Gene) - Inhibitory neurons HBINH8 |
| PDE2A | - CC - St - Hb - Hipp - Med - Pons - Tlm | - Excitatory neurons TEGLU1:24 - D1/D2 medium spiny neurons MSN1:4 - Serotonergic neurons HBSER1:5 - CA1 : Excitatory neurons TEGLU21-24, CA3 : Excitatory neurons TGLU23 - Cholinergic neurons HBCHO1-2 - Afferent nuclei of cranial nerves III-V HBCHO4, Excitatory neurons HBGLU8 - Cholinergic neurons DECHO2 |
| PDE3A | - ENS | - Nitrergic enteric neurons ENT1-2, Enteric mesothelial fibroblasts ENMFB |
| PDE3B | - Med - DRG | - Serotonergic neurons HBSER4, Inhibitory neurons HBINH2 - Peripheral Sensory Neurons PSPEP1:4 |
| PDE4A | - Hb - Hipp - CC | - Excitatory neurons HBGLU4:9 - CA1 : Excitatory neurons TEGLU21 - Excitatory neurons TEGLU1:19 |
| PDE4B | - Med - ENS | - Excitatory neurons HBGLU1, Cholinergic neurons HBCH10 - Cholinergic enteric neurons VGLUT2 ENT7 |
| PDE4C | - DRG | - Peripheral Sensory Neurons PSNP6 |
| PDE4D | - Med - ENS - CC | - Noradrenergic neurons HBNOR, Cholinergic neurons HBCHO1, Inhibitory neurons HBINH2 - Cholinergic enteric neurons ENT6 - Excitatory neurons TEGLU2-3-4-10-11 |
| PDE5A | - CC-Hipp - Med - ENS | - R-LM border Cck interneurons TEINH11 - Afferent nuclei of cranial nerves HBCHO3 - Cholinergic enteric neurons ENT1:6 |
| PDE6A | - Pons | - Excitatory neurons HBGLU8 |
| PDE6B | - Tlm | - Neuroblast-like DETPH |
| PDE6C | - Tlm | - Neuroblast-like DETPH |
| PDE6D | - DRG | - Peripheral Sensory Neurons PSNP1:6 |
| PDE6G | - Tlm | - Neuroblast-like DETPH (Marker gene) |
| PDE7A | - Pons | - Excitatory neurons HBGLU6:9 |
| PDE7B | - St | - D2 medium spiny neurons MSN2 |
| PDE8A | - Pons | - Serotonergic neurons HBSER1:5 |
| PDE8B | - CC - St - Med - Pons - DRG | - Excitatory neurons TEGLU5-7-8-10-11-14-15-16 - D1 medium spiny neurons MSN1-4 - Afferent nuclei of cranial nerves HBCHO3 - Excitatory neurons HBGLU8 - Peripheral Sensory Neurons PSNP6 |
| PDE9A | - Med | - Excitatory neurons HBGLU1-8 |
| PDE10A | - St | - D1/D2 medium spiny neurons MSN1-4 (Marker gene of MSN3-4) |
| PDE11A | - DRG - Hyp | - Peripheral Sensory Neurons PSNP1:3, PSPEP2-3-6-8 - Peptidergic neurons HYPEP8 |
| PDE12 | - Med - Hb - DRG - ENS | - Afferent nuclei of cranial nerves HBCHO3 - Serotonergic neurons HBSER2-5 - Peripheral Sensory Neurons PSNP2-3-6 - Cholinergic enteric neurons ENT9 |

CC : Cerebral Cortex

Tlc : Telencephalon

Hyp : Hypothalamus

St : Striatum

Hb : Hindbrain

DRG : Dorsal Root Ganglion

OB : Olfactory Bulb

Med : Medulla

Hipp : Hippocampus

Tlm : Thalamus

ENS : Enteric nervous systems
