# Supplementary material for: Role of phosphodiesterases in the pathophysiology of neurodevelopmental disorders
Source: Mol Psychiatry. 2021 Jan 7;26(9):4570–82. doi: 10.1038/s41380-020-00997-9 (PMC8589663; doi:10.1038/s41380-020-00997-9)
Supplement: Supplementary file 2 — Supplementary Table II [file 41380_2020_997_MOESM2_ESM.docx]

**Supplementary Table II. Highest expression of PDEs in human brain regions**

| Frontal Cortex | PDE2A |
| --- | --- |
| Parietal Cortex | PDE2A |
| Temporal Cortex | PDE2A |
| Hippocampus | PDE2A |
| Caudate Nucleus | PDE1B; PDE10A |
| Substantia Nigra | PDE1C; PDE4B |
| Nucleus Accumbens | PDE1B; PDE2A; PDE10A |
| Cerebellum | PDE4B; PDE9A; PDE10A |
| Talamus | PDE1C; PDE4B |
| Hypothalamus | PDE4B; PDE1C; PDE8B |
| Dorsal root ganglia: | PDE9A; PDE5A; PDE2A; PDE1C |
| Spinal cord | PDE4B |
