# Supplementary material for: Role of phosphodiesterases in the pathophysiology of neurodevelopmental disorders
Source: Mol Psychiatry. 2021 Jan 7;26(9):4570–82. doi: 10.1038/s41380-020-00997-9 (PMC8589663; doi:10.1038/s41380-020-00997-9)
Supplement: Supplementary file 3 — Supplementary Table III [file 41380_2020_997_MOESM3_ESM.docx]

|  | PDE1A | PDE1C | PDE5A | PDE10A | PDE11A |
| --- | --- | --- | --- | --- | --- |
| E11.5 | - | - | + | - | - |
| E13.5 | - | + | + | - | - |
| E15.5 | - | + | - | - | - |
| E18.5 | - | + | - | + | + |
| P4 | + | - | - | + | - |
| P14 | + | + | + | + | - |
| P28 | + | + | + | + | + |

**Supplementary Table III. The expression levels of PDEs in brain during development**

The expression of each PDE was studied at the developmental age as indicated in the Table.

E= embryonal day ; P= post natal day
