# Supplementary material for: Role of phosphodiesterases in the pathophysiology of neurodevelopmental disorders
Source: Mol Psychiatry. 2021 Jan 7;26(9):4570–82. doi: 10.1038/s41380-020-00997-9 (PMC8589663; doi:10.1038/s41380-020-00997-9)
Supplement: Supplementary file 4 — Supplementary Table IV [file 41380_2020_997_MOESM4_ESM.docx]

**Supplementary Table IV. Phenotypes of PDEs mouse models**

**KO: Knockout KD: Knockdown**

| ***Pde1b* KD** | Enhancement of spatial and contextual memory [31] |
| --- | --- |
| ***Pde1b* KO** | Locomotor hyperactivity, spatial learning deficits [32], antidepressant-like phenotype [33] |
| ***Pde2a* KO** | Lethal during embryogenesis [37] |
| ***Pde4b* KO** | Reduction in prepulse inhibition, baseline motor activity and an exaggerated locomotor response to amphetamine [44]  Deficits in associative learning in conditioned fear paradigm [45]  Increased anxiety (reduced time spent in light and exploration) [46] |
| ***Pde4d* KO** | Decreased immobility in tail-suspension and forced-swim test [49] |
| ***Pde10a* KO** | Locomotor hypoactivity and delay in the acquisition of conditioned avoidance responding [50] |
| ***Pde11a* KO** | Enlarged lateral ventricles and increased activity in CA1 [54]  Altered formation of social memories and abnormal stabilization of mood [55] |
