# Supplementary material for: Role of phosphodiesterases in the pathophysiology of neurodevelopmental disorders
Source: Mol Psychiatry. 2021 Jan 7;26(9):4570–82. doi: 10.1038/s41380-020-00997-9 (PMC8589663; doi:10.1038/s41380-020-00997-9)
Supplement: Supplementary file 5 — Supplementary Table V [file 41380_2020_997_MOESM5_ESM.docx]

**Supplementary Table V. Inhibitors of PDEs in recent clinical studies.**

| **PDE** | **Inhibitor** | **Clinical Trial or Retrospective Study** |
| --- | --- | --- |
| PDE2A | TAK-915 | NCT02584569 - Phase I - Pharmacodynamics (2015-2016) |
|  | PF-05180999 | NCT01981499 - Migraine (Unpublished results) - Phase I (2013-2014) |
| PDE3 | Cilostazol | NCT02491268 - AD - Phase II (2015-2019) |
|  |  | Ameliorates mini-mental state examination score - AD [149] (2013) |
|  |  | Reduces decline of cognitive function - AD [75] (2017) |
|  |  | Decreases risk of dementia [74] (2001) |
| PDE4 | Rolipram | NCT00369798 - Depression - Phase I (2006-2018) |
|  | Roflumilast | Improves verbal memory - SCZ [78,79] (2019) |
|  |  | [NCT02051335 - Cognition in old healthy patients](https://clinicaltrials.gov/show/NCT02079844) - Phase I (2014-2017) |
| PDE4D | BPN14770 | NCT02840279-NCT02648672 - Phase I - Pharmacodynamics (2016-2017) |
|  |  | NCT03569631 - Phase II - FXS (2018-2020) |
| PDE9A | PF-04447943 | Do not improve cognition and behavior in AD [97] (2014) |
|  | BI 409306 | Phase I - Pharmacodynamics [150] (2018) |
|  |  | Do not improve cognition in AD [96] (2019) |
|  |  | NCT03351244 - SCZ - Phase II (2017-) |
| PDE10A | TAK-063 | [NCT01892189](https://clinicaltrials.gov/ct2/show/NCT01892189) - Phase I - Psychotic-like Symptoms (2013-2017) |
|  |  | [NCT02477020 - SCZ](https://www-sciencedirect-com.proxy.unice.fr/science/article/pii/S0920996418305401?via%3Dihub) - Phase II (2015-2017) |
|  | Lu AF11167 | NCT03929497 - NCT03793712 - SCZ - Phase II (2019- ) |

**Supplementary References**

149. Taguchi A, Takata Y, Ihara M, Kasahara Y, Tsuji M, Nishino M, et al. Cilostazol improves cognitive function in patients with mild cognitive impairment: a retrospective analysis. Psychogeriatrics. 2013;13:164–169.

150. Moschetti V, Kim M, Sand M, Wunderlich G, Andersen G, Feifel U, et al. The safety, tolerability and pharmacokinetics of BI 409306, a novel and potent PDE9 inhibitor: Overview of three Phase I randomised trials in healthy volunteers. Eur Neuropsychopharmacol. 2018;28:643–655.
